# Bayesian outcome selection modeling

**DOI:** 10.1002/sta4.568

**Published:** 2023-03-29

**Authors:** Khue-Dung Dang, Louise M. Ryan, Richard J. Cook, Tugba Akkaya Hocagil, Sandra W. Jacobson, Joseph L. Jacobson

**Affiliations:** 1 School of Mathematics and Statistics, University of Melbourne, Melbourne, 3010, Australia; 2 School of Mathematical and Physical Sciences, University of Technology Sydney, Sydney, 2007, Australia; 3 Australian Research Council Centre of Excellence for Mathematical and Statistical Frontiers, Melbourne, 3010, Australia; 4 Department of Statistics and Actuarial Science, University of Waterloo, Waterloo, N2L 3G1, Canada; 5 Department of Psychiatry and Behavioral Neurosciences, Wayne State University School of Medicine, Detroit, Michigan, 48201, USA

**Keywords:** Bayesian methods, biostatistics, variable selection

## Abstract

In psychiatric and social epidemiology studies, it is common to measure multiple different outcomes using a comprehensive battery of tests thought to be related to an underlying construct of interest. In the research that motivates our work, researchers wanted to assess the impact of in utero alcohol exposure on child cognition and neuropsychological development, which are evaluated using a range of different psychometric tests. Statistical analysis of the resulting multiple outcomes data can be challenging, because the outcomes measured on the same individual are not independent. Moreover, it is unclear, a priori, which outcomes are impacted by the exposure under study. While researchers will typically have some hypotheses about which outcomes are important, a framework is needed to help identify outcomes that are sensitive to the exposure and to quantify the associated treatment or exposure effects of interest. We propose such a framework using a modification of stochastic search variable selection, a popular Bayesian variable selection model and use it to quantify an overall effect of the exposure on the affected outcomes. The performance of the method is investigated empirically and an illustration is given through application using data from our motivating study.

## 1 | INTRODUCTION

In psychological and social epidemiology studies, participants are typically assessed using a comprehensive battery of tests or tasks designed to measure psychological, neurological, or cognitive outcomes that are difficult to measure directly. Analysts then face the challenge of how to best handle the resulting multiple outcomes. Often, a large number of outcomes are collected, and it can be challenging to decide which outcomes to include in the analysis. Scientists typically rely on previous studies, in combination with expert knowledge, to select the outcomes on which to focus. No statistical framework has been available for identifying outcomes that are sensitive to an exposure, nor has such a framework been developed to quantify the magnitude of effects.

There is a rich literature on statistical methods for the analysis of multiple outcomes data. The simplest approach is to analyze each outcome separately, but such an analysis requires adjustment for multiple comparisons (Lefkopoulou & Ryan, 1993). Structural equation models (SEMs) can also be used to model correlated outcomes by treating the outcomes as manifestations of the latent variables (Budtz-Jørgensen et al., 2002; Dunson, 2000; Sánchez et al., 2005). However, the regression coefficients characterizing the relationship between the exposure and the latent factor can be problematic to interpret, and inference is sensitive to model misspecification (Sammel & Ryan, 2002). Meta-analysis is another popular approach to synthesis of multiple outcomes data, but relatively little work has been carried out for dealing with highly correlated outcomes in observational settings (Akkaya Hocagil et al., 2022; Berkey et al., 1998; Ryan, 2008; van den Noortgate et al., 2015). Generalized estimating equations (Lefkopoulou et al., 1989; Liang & Zeger, 1986) have also been used to analyze multiple outcome data, with working covariance matrices specified to accommodate correlations across outcomes, because the repeated observations on each individual can be viewed as a special type of clustered data. Generalized linear mixed models offer another framework to model the effect of exposure on multiple outcomes (Sammel et al., 1999; Thurston et al., 2009). In this paper, we extend the generalized linear modeling approach for the analysis of multiple outcomes.

A limitation of the available statistical methods for analyzing multiple outcomes data is that researchers must specify the outcomes to be included in the analysis. As mentioned above, this is usually done using expert knowledge or following some gatekeeping procedure to select the subset of affected outcomes (see, for example, Turk et al., 2008). However, this can be challenging when outcomes are high dimensional or when expert knowledge does not provide strong guidance. Moreover, using exploratory data analysis to guide the decision-making increases the risk of distorting statistical inference due to multiple comparisons. We develop and evaluate a principled statistical approach for identification of relevant outcomes on which to model the exposure effects, while accounting for the correlation among the outcomes.

We refer to the challenge of identifying which of many observed outcomes are sensitive to an exposure as the outcome selection problem and show that it can be reframed as a classical variable selection problem. Variable selection is typically carried out to choose a subset of candidate predictors that together explain most of the variation in a single response variable. The variable selection literature has a long history, from earlier frequentist approaches such as “best subset” regression, model selection based on Akaike/Bayesian information criterion (Akaike, 1998; Schwarz, 1978), backward and forward stepwise regression, to the more recent Bayesian methods that involve a wide range of “slab-and-spike” or shrinkage priors; see Hastie et al. (2020), O’Hara and Sillanpää (2009), and van Erp et al. (2019) for some recent reviews. To the best of our knowledge, these ideas and approaches have not been adapted to deal with the setting where one aims to select which outcomes in a large set of candidate outcomes are sensitive to an exposure.

In this paper, we first show how the problem of interest can be reframed as one of variable selection. We adopt a Bayesian approach to analyze outcomes and identify those that are strongly affected by the exposure. The model is motivated by the popular stochastic search variable selection (SSVS) method, but we extend the SSVS prior to allow estimation of a mean effect among the sensitive outcomes. A random effects model is used to account for the correlation among outcomes measured on the same individuals.

The paper is organized as follows: In Section 2, we present the basic model and show how the outcome selection problem can be reframed as one of variable selection. We also discuss the associated computing approach. In Section 3, we assess the performance of our method in comparison to other variable selection models based on a simulation study. In Section 4, we use the model and method to analyze data from our motivating application regarding the effect of in utero alcohol exposure on different measures of child cognition. In Section 5, we present some conclusions and discussion.

## 2 | METHODOLOGY

### 2.1 | Addressing the outcome selection problem

Suppose we observe K continuous outcomes for each of n independent individuals. The outcomes will typically be correlated because they are measures from the same individual, though they may be of different scales and nature. For example, the outcomes may be measuring different domains of a person’s cognitive function (verbal versus mathematical). Therefore, exposure effects are not expected to be exactly the same across affected outcomes but vary around a mean level μ, which we identify as the parameter of interest. For each individual, we observe an exposure value and some other observed predictor variables, which we denote as z. In the application discussed in Section 4, z is a propensity score computed for each individual to adjust for confounders.

We now show how to express our multiple outcomes data as panel data in long format. Consider a sample of n independent individuals labeled j=1,…,n, where each individual has measurements on K outcomes labeled p=1,…,K. For now, assume there are no missing data and that all K outcomes have been measured on all n individuals. Now suppose we stack the observations from all n individuals together, giving us a data set with nK observations in total. Row i of this data set records the observed outcome corresponding to individual j[i] and outcome p[i].

**FIGURE 1:**
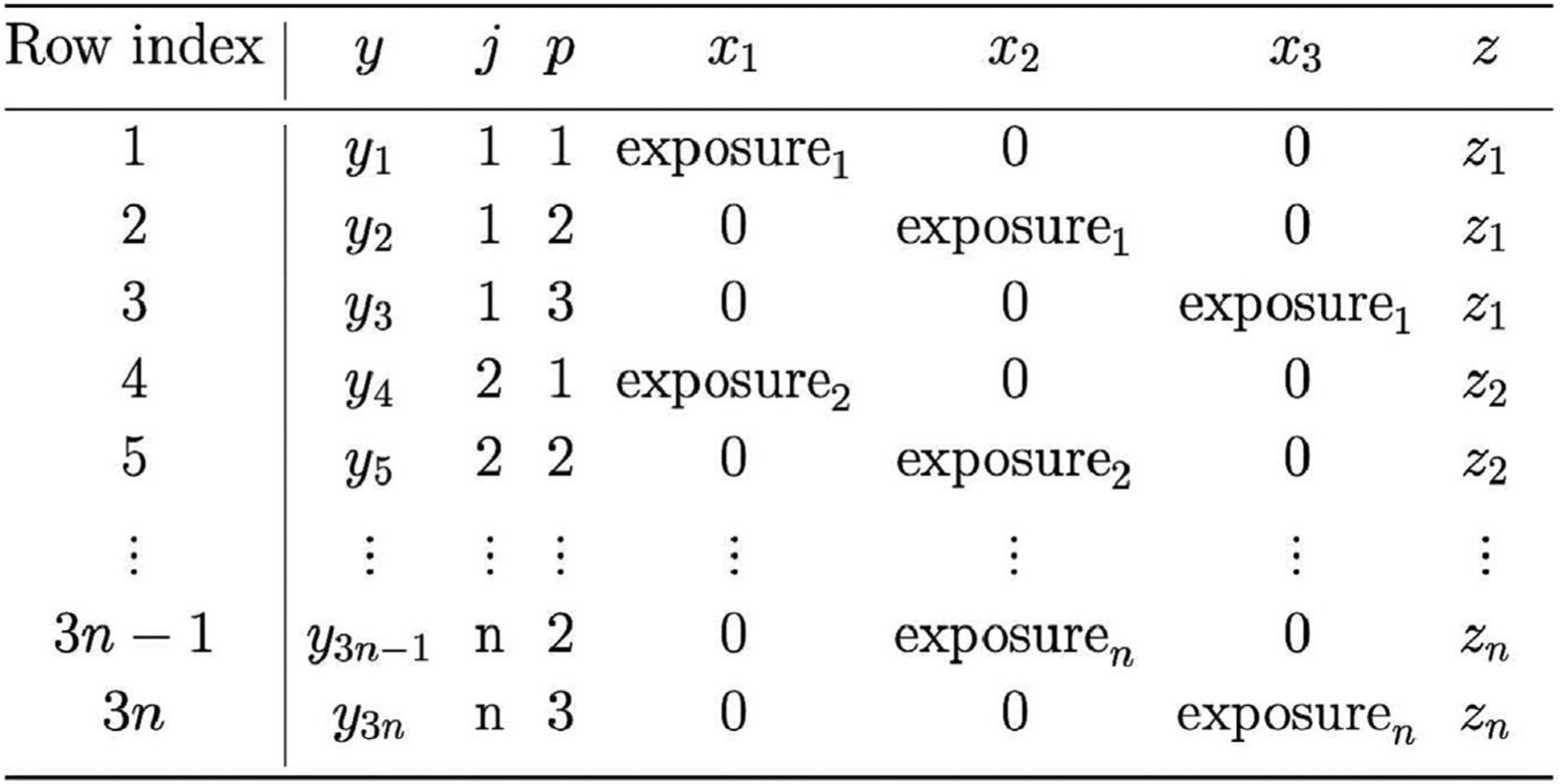
Illustration of a data table with n individuals and K=3 outcome variables.

To represent the multiple outcome problem as a multiple predictors problem, we first define a new set of covariates $x_{k},k=1,\ldots ,K$:

$$x_{k}[i]={\text{exposure}}_{j[i]}1(p[i]=k),$$

for i=1,…,nK. The *i*th value of $x_{k}\left(x_{k}[i]\right)$ is the interaction between the exposure level of individual j[i] and a dummy variable indicating whether the value of the outcome p[i] is k. Including this exposure by outcome interaction term is critical because it allows for a potentially different exposure effect, depending on outcome. An example of the dataframe format and how to map from the multiple outcome format to the stacked format for n individuals and K=3 outcome variables is presented in Figure 1. This “trick” of expressing the multiple outcomes problem in terms of repeated measures has been widely used in the literature, making it straightforward then to analyze multiple outcomes using standard mixed modeling or GEE software (Lefkopoulou et al., 1989).

We will base our analysis on a linear mixed model, as follows:

(2.1) $$y\left[i\right]=\nu_{p\left[i\right]}+\alpha_{j\left[i\right]}+\sum_{k=1}^{K}\beta_{k}x_{k}\left[i\right]+\gamma_{p\left[i\right]}z_{j\left[i\right]}+\epsilon \left[i\right],$$

for i=1,…,nK, individual j=1,…,n and outcome p=1,…,K. The error terms ϵ[i] are independent and normally distributed, $\epsilon [i]\sim N\left(0,\sigma_{pii}^{2}\right)$ and the random effect $\alpha_{j[i]}\sim N\left(0,\sigma_{r}^{2}\right)$ accounts for the within-individual correlation and $\alpha_{j}⊥\alpha_{j^{'}}$ for $j\neq j^{'}$.

The parameters $\nu_{p}$ and $\gamma_{p}$ are outcome-specific intercepts and coefficients for z, and the coefficients $\beta_{k}$ represents the exposure effect on outcome k. In a classical multiple outcomes setting, it is typical to assume that all outcomes are associated in a similar way with the exposure or treatment of interest, with effects varying around a mean level μ. It is natural to assume

$$\beta_{k}\sim N\left(\mu ,\tau^{2}\right),$$

for k=1,…,K, and then assign appropriate priors for μ and τ to estimate the model using a Bayesian estimation procedure. We now want to generalize this framework to allow for the possibility that not all K outcomes are affected by the exposure. Asking the question of which outcomes should be included becomes a problem of variable selection, based on the K covariates $x_{1},\ldots ,x_{k}$. From a modeling perspective, allowing for some of the outcomes to be unaffected by the exposure simply corresponds to setting $\beta_{k}=0$ for those variables.

Variable selection methodologies have been extended to deal with random effects; see for example Bondell et al. (2010), Fan and Li (2012), and Yang et al. (2020). For our model, because we do not need to perform variable selection for the random effects, it is straightforward to use existing techniques for the independent predictors $x_{k}$. This means that we can potentially use any of a variety of sparsity priors, such as SSVS (George & McCulloch, 1993), Bayesian LASSO (Figueiredo, 2003; Park & Casella, 2008), and the horseshoe prior (Carvalho et al., 2009) for outcome selection. However, because we are interested in selecting the subset of affected outcomes variables and quantifying the mean exposure effect on these variables, in this study we focus on the use of the SSVS method. We discuss this further in the next section.

### 2.2 | Stochastic search variable selection

There is a large literature on Bayesian variable selection methods; however, in this paper, we only discuss the “slab and spike” type of priors, as they are suitable for our problem of identifying sensitive outcomes from a large number of outcomes. Methods that compare models by Bayes Factor (Kass & Raftery, 1995) or criteria such as DIC (Spiegelhalter et al., 2002) or WAIC (Watanabe & Opper, 2010) require fitting all candidate models and hence only applicable when comparing a small number of models. Therefore, they are not suitable for the outcome selection problem.

Methods involving a “slab and spike” prior can be divided, broadly, into two categories: Methods that specify a prior that approximate the “slab and spike” shape for the coefficients $\beta_{k}$; and methods that use latent indicator variables that indicate whether a covariate is included in the model. Shrinkage priors such as the Bayesian LASSO (Figueiredo, 2003; Park & Casella, 2008) and the horseshoe prior (Carvalho et al., 2009) belong to the first category. The implementation of these methods is straightforward and they have had extensive use in recent years. However, it is not clear how to modify these priors to incorporate a common mean of the nonzero coefficients. Yang et al. (2020) proposed using SSVS for selection of fixed effects in linear mixed models; however, they also did not consider estimating the mean effect.

The second category of approach defines a latent variable $I_{k}$ that indicates whether a coefficient $\beta_{k}$ is nonzero. In the approaches proposed by Kuo and Mallick (1998) and Dellaportas et al. (2002), a coefficient $\beta_{k}$ is set to 0 if $I_{k}=0$. Both methods specify $\beta_{k}=I_{k}\theta_{k}$ and hence require an appropriate prior for $\theta_{k}$. These approaches can be challenging to tune to ensure that the iterates of $I_{k}$ do not get stuck at 0 or 1. For example, mixing may be poor for the Kuo and Mallick (1998) approach if the prior for $\theta_{k}$ is too vague (O’Hara & Sillanpää, 2009). We can assume $\theta_{k}\sim N\left(\mu ,\tau^{2}\right)$ with unknown μ and τ, but the model will be hard to fit and we cannot interpret μ as the mean of all $\beta_{k}$ of which $I_{k}=1$.

Our method is motivated by the SSVS method (George & McCulloch, 1993), which defines a mixture prior for $\beta_{k}$ instead: Let $l_{k}$ be a latent indicator variable, with $I_{k}=1$ means covariate k is included in the model, $I_{k}=0$ means it is not. The indicator affects the prior of $\beta_{k}$, so we can define a joint prior for $\left(I_{k},\beta_{k}\right)$ as

$$p\left(I_{k},\beta_{k}\right)=p\left(\beta_{k}|I_{k}\right)p\left(I_{k}\right).$$

Conditioning on $I_{k}$, the prior of $\beta_{k}$ is

$$p\left(\beta_{k}|I_{k}\right)=\left(1-I_{k}\right)N\left(0,g_{1}\right)+I_{k}N\left(0,\tau^{2}\right).$$

For our outcome selection framework, we propose to modify the SSVS prior to incorporate the mean exposure effect μ on the sensitive outcomes. Conditioning on $I_{k}$, we now have a mixture prior for $\beta_{k}$

(2.2) $$p\left(\beta_{k}|I_{k}\right)=\left(1-I_{k}\right)N\left(0,g_{1}\right)+I_{k}N\left(\mu ,\tau^{2}\right).$$

To improve the performance of the model, we follow Meuwissen and Goddard (2004) and modify the prior in (2.2) to

(2.3) $$p\left(\beta_{k}|I_{k}\right)=\left(1-I_{k}\right)N\left(0,\tau^{2}/c\right)+I_{k}N\left(\mu ,\tau^{2}\right).$$

The tuning parameter c should be chosen to ensure good separation between the “in” and “out” variables. The standard deviations $\tau ,\sigma_{k}$, and $\sigma_{r}$ are assigned log-normal priors in our simulation study and application.

Note that the posterior mean of $l_{k}$ will be the posterior probability that outcome k is included in the model; hence, it will be important in terms of interpreting the results of our model fit. The prior, $p\left(I_{k}=1\right)$, can simply be a categorical distribution with a fixed probability parameter. This prior probability may be different across outcomes, based on the experts’ knowledge, or fixed at 0.5 so that the prior is non-informative. The prior probability $p\left(I_{k}=1\right)$ can also be treated as a parameter to be estimated (O’Hara & Sillanpää, 2009). For the examples in this paper, we simply set a prior probability $p\left(I_{k}=1\right)$ for each k.

For the examples in this paper, an outcome is classified as “relevant” if the posterior mean of the corresponding $I_{k}$ is greater than 0.5. We note that the threshold may affect the conclusion on the relevance of each outcome variable but does not change the estimate of the mean effect μ. Of course, other thresholds could be used. We suggest that it is best to report the posterior probabilities of $I_{k}=1$ for all k. An alternative approach is to look at the whole vector I to identify the most frequently sampled subsets of outcomes.

## 3 | SIMULATION STUDY

### 3.1 | Setup

In this section, we demonstrate the performance of the method using simulated data. The aim of this simulation study is to assess the performance of our prior for outcome selection for different effect sizes. In the exercise, we set the number of outcomes to K=20 and a moderate sample size of n=100. We examine the performance of the proposed model with different numbers of relevant outcomes $K_{1}=5,10,15$.

We simulated 10 data sets from the model (2.1) described in Section 2.1. We set the parameters value to generate the data sets as follows: The intercepts $\nu_{k}$ values are randomly picked from N(0,1) and standard deviations $\sigma_{k}^{2}$ are generated from N(1.5,0.3) for k=1,…,20. The coefficients $\beta_{k}$ corresponding to the relevant outcomes were generated from $N\left(\mu ,0.01\mu^{2}\right)$ to ensure that the $\beta_{k}$ are scattered closely enough around μ. We used two different values for the mean common effect μ:μ=-0.1 and μ=-3.

Given these “true” parameters values, in each simulation, we create a data set by first generate the exogenous variable $z_{j[i]}$ from N(0,1) and exposure ${\;}_{j[i]}|z_{j[i]}\sim 1\left(z_{j[i]}<0\right)N\left(0,0.5^{2}\right)+\left(1-1\left(z_{j[i]}<0\right)\right)N(1,1)$ and then generate the outcome y according to model (2.1).

We then fit model (2.1) using the prior in (2.3) with c=100 to each of the 10 data sets. We examine both versions of SSVS: Our proposed model in which μ is a parameter and the standard SSVS prior where μ=0. The prior probability of $I_{k}$ is $p\left(l_{k}=1\right)=0.5$ for all outcome k. For comparison, we also fit the model where $\beta_{k}$ are assumed a hierarchical prior $\beta_{k}\sim N\left(\mu ,\tau^{2}\right)$ with unknown μ and $\tau^{2}$. We also fit the model that only uses the correct relevant outcomes, assuming $\beta_{k}\sim N\left(\mu ,\tau^{2}\right)$. We call this the “subset model.” The result of the subset model is treated as the “standard” because it is the model that uses the correct set of outcomes.

The rest of the parameters are assigned fairly flat priors. For example, we use a normal N(0,100) prior for $\mu ,\nu_{k}$ and $\gamma_{k}$. The parameters $\sigma_{r}$ and $\sigma_{k},k=1,\ldots ,K$ are assigned log-normal(0,10) priors. In all models, τ is assigned a log-normal(0,1) prior.

For each simulation, we record the number of outcomes identified as relevant, the number of correctly identified outcomes, the number of false positives, and the estimated μ. An outcome is classified as “relevant” if the posterior mean of the corresponding $I_{k}$ is greater than 0.5. The results presented here represent the average over the 10 simulations for each setting. The SSVS models are fitted using the software JAGS (Plummer, 2003) and the other models are implemented with STAN (Carpenter et al., 2017).

Note that in this simulation study, the first setting with μ=-0.1 represents a situation when the effect is weak with small data, so that the posterior standard deviation is large. In this case, it would be difficult for the model to decide whether a $\beta_{k}$ is 0 or not. The second setting μ=-3 mimics the situation in which the effect is stronger, and the selection method is expected to work better.

### 3.2 | Results

The performance of the SSVS algorithm in detecting the affected outcomes for different μ and $K_{1}$ is presented in Table 1. The results suggest that the original and our modified SSVS algorithms have very similar performance, though neither do well in detecting the affected outcomes when μ is small. This is expected as the overall effect μ is small, so some of the relevant $\beta_{k}$ would be close to 0. Because here we used uninformative priors for $l_{k}$, the algorithm will keep switching between stage $I_{k}=0$ and $l_{k}=1$ for these outcomes. This is similar to the phenomenon observed by O’Hara and Sillanpää (2009), where the posterior probabilities of $l_{k}$ are close to 0.5 and some outcomes are classified incorrectly by chance.

Table 2 shows the mean squared errors of estimating the individual coefficient $\beta_{k}$:

(3.1) $$\text{MSE}=\frac{1}{K}\sum_{k=1}^{K}{\left({\hat{\beta }}_{k}-\beta_{k}\right)}^{2},$$

where we take the estimate ${\overset{ˆ}{\beta }}_{k}$ to be the posterior mean of $\beta_{k}|l_{k}=1$ if the posterior mean of $I_{k}$ is greater than 0.5; otherwise we set ${\overset{ˆ}{\beta }}_{k}=0$. For both large and small values of μ, the SSVS priors provide more accurate estimates of $\beta_{k}$ in terms of MSE, compared to the model without variable selection.

Lastly, Table 3 shows the estimated of μ, averaged over 10 simulations, by different priors. Table 3 shows that our modified SSVS can provide estimates of μ that are closer to the result from the subset model, especially for large μ. However, when the effect is weak, the model is not able to estimate μ accurately because it fails to identify the correct set of sensitive outcomes.

The simulation example shows that SSVS priors can provide accurate estimates of the coefficients and accurately identify the affected outcomes and estimate the mean effect when μ is far from 0. However, it may require more informative priors for $I_{k}$ and better tuning to capture small effects accurately. The R code for the study is provided on Github–see https://github.com/khuedung91/BayesianOutcomeSelection/.

## 4 | EFFECT OF PRENATAL ALCOHOL EXPOSURE ON CHILDREN IN DETROIT, MICHIGAN

In this section, we apply our proposed framework to data collected as part of an investigation of the long-term effect of prenatal alcohol exposure (PAE) on a child’s cognitive and behavioral function. Numerous studies have shown that high levels of PAE can result in a distinct pattern of craniofacial anomalies, growth restriction, and cognitive and behavioral deficits, a condition known as fetal alcohol syndrome (FAS) (Hoyme et al., 2005; Hoyme et al., 2016), the most severe of a continuum of fetal alcohol syndrome disorders (FASD) (Carter et al., 2016; Jacobson et al., 2004; Jacobson et al., 2008; Mattson et al., 2019). Alternatively, some individuals with PAE exhibit cognitive and/or behavioral impairment without the characteristic craniofacial dysmorphology and/or growth restriction, a disorder known as alcohol-related neurodevelopmental disorder (ARND).

Our data come from a longitudinal study, funded by the US National Institutes of Health and conducted in Detroit, Michigan. In this study, the mothers were interviewed prenatally about their alcohol consumption during pregnancy, and the children were followed throughout childhood, many of them up until they were 20 years of age. The study collected a large number of variables reflecting responses on various neuro-cognitive tests and behavioral outcomes assessed on the children throughout childhood. Each of the administered tests could be classified as relevant to one of several different domains including cognition, executive function, and behavior, among others. Previous neurocognitive studies have suggested that the impact of PAE on all of these domains will not be the same, given that alcohol may have a stronger effect on certain parts of the brain, while other areas may be relatively unaffected or spared, depending on the timing, genetic vulnerability, and ethnic or racial group of the exposure (Jacobson et al., 2004; Jacobson & Jacobson, 1999; 2002). Recent analyses by our group made use of expert knowledge to select outcomes for analysis and simply assumed that each had been affected by PAE to some extent (Jacobson et al., 2021).

To illustrate our methodology, in this paper we focus on a set of 14 outcomes collected when the children were approximately 7 years of age. The first eight outcomes come from the Achenbach Child Behavior Checklist (CBCL) and Teacher’s Report Form (TRF) at age 7 (Achenbach, 1991). The CBCL is a checklist completed by the parent and designed to detect emotional and behavioral problems in children and adolescents, whereas the TRF represents the child’s principal teacher’s report of the similar. These assessments include the child’s internalizing and externalizing behaviors, and social and attention problems. The remaining six outcomes correspond to the results of various cognitive and neuro-developmental tests related to IQ assessed on the Wechsler Intelligence Scales for Children–III (Wechsler, 1991), academic achievement in reading and arithmetic, learning and memory abilities, and executive function. Recent analyses have reported that, of these 14 outcomes, the first eight are relatively less affected by PAE, whereas the last six are more sensitive to alcohol exposure (Jacobson et al., 2021). After preprocessing, the data include outcomes from 336 children. PAE is computed based on the mother’s average daily dose of absolute alcohol consumed (in ounces) during pregnancy (AA/day). Because the distribution of alcohol exposure is positively skewed with a minimum level 0, we compute log(AA/day + 1) and use this as the measure of PAE in the analysis.

### 4.1 | Model and setup

We fit the model (2.1) with the prior in (2.3) to the data set. To adjust for confounders associated with both alcohol exposure and cognitive function, we add a propensity score z, which was computed beforehand. For details on the covariates included in the propensity score and how it was constructed, we refer readers to Akkaya Hocagil et al. (2021). Before running the analysis, we rescaled all outcomes to have mean 0 and variance 1.

We fit our proposed model with a few different settings. We start with an uninformative prior for the indicator $I_{k}$ and set $p\left(l_{k}=1\right)=0.5$ for all k. We use the prior in (2.3) with c=100 for $\beta_{k}$ where τ is assigned a log-normal (0,1) prior. As a comparison, we also try the prior in (2.2) where we fix $g_{1}=0.2^{2}$ and a shrinkage prior. For the shrinkage prior, we simply follow Figueiredo (2003) and assign a Laplace(0,1) prior for the $\beta_{k}$. We also attempt the horseshoe prior (Carvalho et al., 2009) for $\beta_{k}$ but the MCMC has convergence issue and hence the result is not presented here.

To assess how sensitive the result is to the prior probability $p\left(I_{k}=1\right)$, we also fit the model with a more informative set of $p\left(I_{k}=1\right)$,

$$p{\left(I_{k}=1\right)}_{k=1:14}=(0.5,0.5,0.2,0.5,0.8,0.8,0.2,0.8,0.5,0.5,0.8,0.8,0.8,0.5).$$

These prior probabilities were chosen by utilizing expertise knowledge and set the probability of the outcomes that are known to be relevant to be closer to 1. In practice, more informative priors may help the MCMC to have better mixing.

We also fit the model (2.1) to only those outcomes chosen by our SSVS model with a hierarchical prior $\beta \sim N\left(\mu ,\tau^{2}\right)$. We call this model the “subset” model. Similar to in the simulation study, we will compare the estimates of $\beta_{k}$ and μ from this reduced model with our approach.

We assigned a normal N(0,1) prior for μ. We found the appropriate prior’s parameters by fitting model (2.1) to the data using the R package *Ime4*. The estimates of $\beta_{k}$ from *Ime4* suggested that the average effect on the affected outcomes may be around −1, and therefore we used the prior that covers this value. For the rest of the parameters, we chose diffuse priors. The prior for $\sigma_{r}$ and $\sigma_{k},k=1,\ldots ,14$ is log-normal(0,10). The prior for $\nu_{k}$ and $\gamma_{k}$ are normal N(0,1000) and normal N(0,100), respectively. The SSVS models were fitted using the software JAGS (Plummer, 2003), running three chains each with 200,000 burn-in and 200,000 samples with thinning = 10. The other models were implemented in STAN (Carpenter et al., 2017).

### 4.2 | Results

The results are presented in Tables 4 and 5. Table 4 shows the mean posterior probability of $I_{k}=1$. For the Laplace shrinkage prior, we report whether 0 is outside of the 95% credible intervals of the parameters. The table shows that all SSVS models choose the same set of relevant outcomes in different settings. The informative prior on $p\left(I_{k}\right)$ results in different posterior mean of $I_{k}$; however, it does not affect the inference on the outcomes’ relevance for most outcome variables. The only exception is CBCL Externalizing at age 7, of which the posterior probability of being affected is slightly less than 0.5 (0.436 and 0.496) when using a noninformative prior and slightly higher than 0.5 (0.530) when using an informative prior. These results are also similar to that of the Laplace shrinkage prior; however, this prior shrinks more $\beta_{k}$ toward 0 than the SSVS priors.

Table 5 presents the estimates of $\beta_{k}$ and overall effect μ. The SSVS with informative $p\left(l_{k}=1\right)$ and the subset model suggest a strong negative effect of PAE on the cognitive outcomes. These findings are consistent with those in Jacobson et al. (2021). The estimate of τ is similar for both models (0.187 vs. 0.172). On the other hand, the SSVS models with the noninformative prior suggest a weaker effect (−0.324 and −0.303 versus −0.398). The noninformative prior also results in larger estimates of τ (0.219 and 0.233 vs. 0.187).

Table 5 shows that all SSVS models produce smaller estimates for the coefficients of the affected outcomes and hence μ, compared with the subset model. The result here is consistent with our observation in the simulation study in Section 3. However, as shown in Tables 4 and 5, our proposed model produces very similar estimates of $\beta_{k}$ compared with the Laplace prior in all settings. Table 5 also indicates that the informative prior for the indicator $I_{k}$ produces estimates of $\beta_{k}$ and μ that are closer to the subset model that only includes the affected outcomes.

## 5 | CONCLUSION

In this paper, we propose a statistical method for identifying outcomes from a large number of observed variables that are directly affected by an exposure variable. Our method is an extension of standard Bayesian variable selection models to multiple outcomes data, which also provides an estimate of the overall effect of the exposure variable in the subset of affected outcomes. We demonstrate the performance and limitations of our method in a simulation exercise and a real data application.

Our application in modeling the effect of PAE on cognition identified a set of neurodevelopmental tests that are significantly affected by fetal alcohol exposure. In addition, the model indicates a negative overall effect of PAE on the sensitive outcomes. A limitation of the current model is that we only use an individual random intercept to capture the correlations among the outcomes. This approach may not be ideal, and we may consider a more sophisticated correlation structure in future work.

Finally, the proposed framework is shown to be effective in identifying sensitive outcomes in various scenarios. However, it may underestimate the outcome-specific effect size and mean effect when the effects are mild. This is a common issue with variable selection priors; we expect that the result can be improved by using more informative priors for the indicators $I_{k}$.

## Figures and Tables

**TABLE 1:** The average number of outcomes correctly identified as relevant and incorrectly chosen as relevant in different settings with data generated from (2.1). The table shows the results from the original SSVS prior with μ=0 and our proposed prior where μ is unknown. $K_{1}$ is the true number of relevant outcomes. The fourth and fifth columns show the number of outcomes that each model detects as relevant. The next two columns show the number of relevant outcomes correctly identified by each model. The last two columns show the number of irrelevant outcomes that were detected as relevant. All numbers are averaged over 10 simulations.

		# identified as relevant		# correctly identified		# incorrectly identified	
True *μ*	*K*_1_	*μ* unknown	*μ* = 0	*μ* unknown	*μ* = 0	*μ* unknown	*μ* = 0
−0.1	5	4.9	4.4	1.6	1.5	3.3	2.9
	10	5.2	5.3	1.9	2.1	3.3	3.2
	15	5.1	4.4	4.5	3.5	0.6	0.9
−3	5	5	5	5	5	0	0
	10	10	10.1	10	10	0	0.1
	15	15	15	15	15	0	0

**TABLE 2:** The mean squared errors of the models with different effect sizes, in simulated data study. The result is averaged over 10 simulations.

	SSVS – *μ* unknown		SSVS - *μ* = 0		No variable selection	
*K*_1_	Small *μ*	Large *μ*	Small *μ*	Large *μ*	Small *μ*	Large *μ*
5	0.012	0.004	0.007	0.007	0.032	0.066
10	0.022	0.017	0.014	0.032	0.036	0.075
15	0.014	0.032	0.011	0.053	0.036	0.073

**TABLE 3:** Estimates of μ in different settings of the simulated data study in Section 3. The table shows the average of the posterior mean of μ, averaged over 10 data sets, in different μ and $K_{1}$. The standard errors are in brackets. The first column is the true μ. The subset model is the model that used only the correct set of relevant outcomes.

True *μ*	*K*_1_	SSVS	No selection	Subset model
−0.1	5	−0.025 (0.066)	−0.120 (0.145)	−0.209 (0.143)
	10	0.046 (0.069)	0.034 (0.157)	−0.015 (0.169)
	15	−0.026 (0.091)	−0.059 (0.188)	−0.077 (0.213)
−3	5	−3.281 (0.040)	−0.908 (0.150)	−3.365 (0.144)
	10	−2.949 (0.098)	−1.393 (0.152)	−2.875 (0.175)
	15	−2.678 (0.115)	−2.002 (0.210)	−2.688 (0.221)

**TABLE 4:** Summary of $I_{k}$ for different models for Detroit data. For SSVS, the table shows the posterior means of $I_{k}$ for the different outcomes. The table highlights in bold the variables selected by the SSVS prior. For the other method, we report whether 0 is outside the 95% credible interval of the corresponding $\beta_{k}$. The CBCL and TRF tests came from (Achenbach, 1991); the IQ tests were based on the Wechsler Intelligence Test for Children-III (Wechsler, 1991).

		$p\left(I_{k}=1\right)=0.5$		Informative $p\left(I_{k}\right)$ $g_{1}=0.2^{2}$
	Laplace prior	$g_{1}=\tau^{2}/100$	$g_{1}=0.2^{2}$	
CBCL Social Problem	0	0.286	0.384	0.374
**CBCL Attention Problem**	0	0.505	0.533	0.576
CBCL Internalizing	0	0.195	0.247	0.057
CBCL Externalizing	0	0.436	0.496	0.530
**TRF Social Problem**	1	0.856	0.741	0.947
**TRF Attention Problem**	1	0.873	0.742	0.947
TRF Internalizing	0	0.208	0.267	0.065
**TRF Externalizing**	1	0.864	0.746	0.947
Verbal IQ	0	0.291	0.393	0.388
Performance IQ	0	0.345	0.432	0.442
**Freedom from distractibility**	1	0.966	0.809	0.969
**Verbal fluency**	0	0.670	0.629	0.900
**Digit span backwards**	1	0.871	0.737	0.945
Story memory	0	0.267	0.360	0.334

**TABLE 5:** Posterior means of $\beta_{k}$ and μ based on the Detroit data. For the SSVS methods, we report the mean of $\beta_{k}|I_{k}=1$ if the posterior mean of $I_{k}$ exceeds 0.5 and 0 otherwise.

		$p\left(I_{k}=1\right)=0.5$		Informative $p\left(I_{k}\right)$ $g_{1}=0.2^{2}$	
	Laplace prior	$g_{1}=\tau^{2}/100$	$g_{1}=0.2^{2}$		Subset
CBCL Social Problem	−0.090	0.000	0.000	0.000	
**CBCL Attention Problem**	−0.230	−0.274	−0.268	−0.339	−0.475
CBCL Internalizing	0.083	0.000	0.000	0.000	
CBCL Externalizing	−0.195	0.000	0.000	−0.324	
**TRF Social Problem**	−0.486	−0.404	−0.412	−0.460	−0.612
**TRF Attention Problem**	−0.464	−0.396	−0.404	−0.451	−0.602
TRF Internalizing	0.064	0.000	0.000	0.000	
**TRF Externalizing**	−0.499	−0.407	−0.417	−0.464	−0.617
Verbal IQ	−0.112	0.000	0.000	0.000	
Performance IQ	−0.141	0.000	0.000	0.000	
**Freedom from distractibility**	−0.561	−0.447	−0.468	−0.505	−0.622
**Verbal fluency**	−0.327	−0.332	−0.330	−0.388	−0.525
**Digit span backwards**	−0.453	−0.389	−0.400	−0.446	−0.574
Story memory	−0.061	0.000	0.000	0.000	
*μ*		−0.324	−0.303	−0.398	−0.572
τ		0.219	0.233	0.187	0.172
